# An Experimental Study to Evaluate the Role of Buprenorphine, Morphine, and Naltrexone in Animal Models of Depression

**DOI:** 10.7759/cureus.99753

**Published:** 2025-12-21

**Authors:** Nichwa Leuwenhoek Suja Nongtdu,, Veena Verma

**Affiliations:** 1 Pharmacovigilance and Clinical Research, Bharat Serums and Vaccines Limited, Navi Mumbai, IND; 2 Pharmacology and Therapeutics, Vardhman Mahavir Medical College and Safdarjung Hospital, New Delhi, IND

**Keywords:** depression, kappa opioid receptors, morphine, naltrexone, buprenorphine

## Abstract

Background: Opioids have a long-standing history of being used for the treatment of mood disorders, with recent attention directed towards kappa opioid receptors (KOR) for antidepressant drug development. Activation of KOR produces dysphoria and anhedonia, whereas antagonism confers antidepressant-like effects. Buprenorphine, a partial mu-opioid receptor (MOR) agonist and KOR antagonist, exhibits antidepressant properties but carries a risk of abuse liability. Naltrexone, a non-selective opioid antagonist, may mitigate this risk while preserving KOR antagonism. This study evaluated the antidepressant-like effects of buprenorphine, morphine, and naltrexone individually, as well as the combination of buprenorphine and morphine with naltrexone, in validated models of depression.

Experimental approach: Male mice were subjected to unpredictable chronic mild stress (UCMS) for four weeks. Following stress induction, drug treatments were administered for 14 days: buprenorphine (1 mg/kg), naltrexone (1 mg/kg), morphine (5 mg/kg), their combinations, or fluoxetine (10 mg/kg) as a reference. Behavioral assessments included the Sucrose Preference Test (SPT), Open Field Test (OFT), Forced Swim Test (FST), and Tail Suspension Test (TST).

Results: UCMS induced anhedonia and behavioral suppression, confirmed by reduced sucrose consumption, locomotor activity, and increased immobility. Buprenorphine significantly reversed these effects across all assays, with efficacy comparable to fluoxetine. The buprenorphine-naltrexone combination also produced significant antidepressant-like effects, though slightly less pronounced than buprenorphine alone. Morphine and naltrexone showed partial or inconsistent effects.

Conclusion: Buprenorphine demonstrated robust antidepressant-like activity in UCMS, likely mediated by KOR antagonism with contributions from MOR, delta-opioid, and nociceptin opioid peptide (NOP) receptors. Co-administration with naltrexone preserved efficacy while potentially reducing abuse liability. These findings highlight buprenorphine-based regimens as mechanistically novel candidates for further evaluation in treatment-resistant depression.

## Introduction

Major depressive disorder (MDD) remains a leading cause of global disease burden, and despite the availability of numerous antidepressant classes, up to one-third of patients exhibit inadequate response or treatment resistance [1, 2]. Current therapies primarily target monoaminergic systems, which are often insufficient to address the complex neurobiology of depression [3, 4]. This has driven the search for novel agents with faster onset and improved efficacy.

Substantial evidence supports the theory that the opioid system may have a role in depression [5, 6]. Endogenous opioid peptides and associated receptors are expressed in high concentrations in limbic and cortical brain regions implicated in the regulation of behavioral functions, including emotion, mood, motivation, and reward processing [6]. Among the opioids and their receptors, kappa opioid receptors (KORs) have recently been a focus for antidepressant drug development. In particular, activation of the KOR produces anhedonia, dysphoria, and stress-like behavioral states [7]. Conversely, KOR antagonists demonstrate robust antidepressant- and anxiolytic-like effects in rodent models, supporting KOR blockade as a mechanistically distinct therapeutic strategy [8].

Buprenorphine, a semi-synthetic opioid, is of particular interest in this context. It acts as a partial mu-opioid receptor (MOR) agonist and a KOR antagonist, with additional partial agonist activity at the nociceptin/orphanin FQ (NOP; ORL1) receptor [9]. Clinical observations suggest buprenorphine has mood-enhancing properties, and preclinical studies demonstrate antidepressant-like effects across validated behavioral paradigms [10-12]. However, notably, there is a lack of placebo-controlled studies on opioid agonists for the treatment of depression. Recent clinical trials have shown promising results, with ultra-low doses of buprenorphine significantly reducing suicidal ideation after just four weeks of treatment [13]. However, its MOR-agonist activity raises concerns regarding abuse potential. Naltrexone, a relatively nonselective opioid receptor antagonist with higher affinity for MOR than KOR, has been proposed as a strategy to mitigate buprenorphine’s abuse liability while preserving its KOR-mediated antidepressant effects [14]. This pharmacological combination may therefore offer a unique balance of efficacy and safety.

In this study, we have examined whether the opioid agonist or antagonist alone or in combination is effective in mediating the antidepressant-like activity in experimental animal models of depression and whether these drugs can overcome the shortcomings of the existing drugs in the treatment of depression. Therefore, the present study was designed to evaluate the antidepressant-like effects of buprenorphine, naltrexone, morphine, and their combinations in mice subjected to the unpredictable chronic mild stress (UCMS) paradigm, a well-established and translationally relevant model of depression. Behavioral outcomes were assessed using sucrose preference, open-field activity, forced swim, and tail suspension tests. We hypothesized that buprenorphine, alone and in combination with naltrexone, would reverse UCMS-induced behavioral deficits, with efficacy comparable to fluoxetine, and that the addition of naltrexone would enhance KOR antagonism while reducing MOR-mediated liability.

## Materials and methods

The study was conducted in the Department of Pharmacology, Vardhman Mahavir Medical College & Safdarjung Hospital (VMMC & SJH), New Delhi, India, after approval from the Institutional Animal Ethics Committee. All the procedures in the study were done in accordance with the Committee for the Control and Supervision of Experiments on Animals (CCSEA) guidelines, which come under the Department of Animal Husbandry and Dairying (DAHD), Ministry of Fisheries, Animal Husbandry and Dairying (MoFAH&D), constituted under the Prevention of Cruelty to Animals (PCA) Act, 1960. The approval number was IAEC/VMMC/2018/05, dated 10/08/2018.

Swiss Albino male mice weighing between 22 and 25 g were used for the study. The animals were procured from the National Institute of Biologicals (NIB), Noida, India, and were housed in standard laboratory conditions (12-hour light/dark cycle, 21±1°C, and relative humidity of 55±5%), with free access to food and water. Following an acclimatization period of seven days, the mice were randomly assigned to different experimental groups that consisted of six to eight mice per group. Each animal was used only once in the experimental procedures. All the experiments were performed between 0900 hours and 1500 hours.

Drugs and chemicals

Buprenorphine was purchased from Neon Laboratories Limited, New Delhi, India. Morphine, naltrexone, fluoxetine, and normal saline (NS) (0.9% sodium chloride (NaCl) solution in distilled water) were procured from the departmental drug store, VMMC & SJH, New Delhi. All the drugs were freshly prepared with distilled water and were administered intraperitoneally unless indicated otherwise.

Sample size

Six male Swiss Albino mice per group for eight groups were considered an appropriate sample size as per the “resource equation method” for animal studies [15]. According to this method, a value “E” is measured, which is the degree of freedom of analysis of variance (ANOVA). The value of E should lie between 10 and 20. E can be measured by the following formula: E = Total number of animals−Total number of groups. In our study, E = (6x8)-8 = 48-8 = 40, which is more than 20; hence, the sample size in this experiment is more than necessary.

Experimental design and procedure

Animals were randomly allocated into different groups as shown in Table 1, each group comprising six animals. After one week of acclimatization, animals were subjected to the UCMS test [16] for four weeks, followed by two-week treatment periods before exposing them to behavioral testing. Tests included the Sucrose Preference Test (SPT), Open Field Test (OFT), Forced Swim Test (FST), and Tail Suspension Test (TST) [17-20].

**Table 1:** Different treatment groups The final doses of each drug were selected on the basis of a pilot study. UCMS: unpredictable chronic mild stress

Groups	Drugs/Placebo
A	Normal control (without exposure to UCMS, normal saline 0.1-0.5 ml, ip)
B	Stressed control (with exposure to UCMS, normal saline 0.1-0.5 ml, ip)
C	Fluoxetine 10 mg/kg, ip
D	Buprenorphine 1 mg/kg, ip
E	Morphine 5 mg/kg, ip
F	Naltrexone 1 mg/kg, ip
G	Morphine 5 mg/kg + Naltrexone 1 mg/kg, ip
H	Buprenorphine 1 mg/kg + Naltrexone 1 mg/kg, ip

Methodology

UCMS

The UCMS procedure was performed as described previously with minor modifications [16]. The stressor regime includes low-grade stressors such as soiled cages, tilting of the cage, alterations of the light-dark cycle, periods of food or water deprivation, grouping, etc. (Table 2). The animals were exposed to two stressors simultaneously in a random manner for a short time (a few hours to a day) over a period of four weeks. The animals were exposed to the stressors throughout the entire circadian period (i.e., during the dark and the light periods) and randomly (applying the different stressors in an unpredictable manner). To avoid any habituation of the animals to the stressors and to make the protocol more unpredictable, the order of stressors was changed weekly during the stress exposure period.

**Table 2:** UCMS protocol for inducing depression UCMS: unpredictable chronic mild stress; hrs: hours

Day	Stressor 1	Stressor 2
Monday	Cage tilting (24 hrs: 9 AM-9 AM)	Water deprivation (24 hrs: 9 AM-9 AM)
Tuesday	Food deprivation (24 hrs: 9 AM-9 AM)	Dark room (24 hrs: 9 AM-9 AM)
Wednesday	Predator smell (24 hrs: 9 AM-9 AM)	Overnight illumination (24 hrs: 9 AM-9 AM)
Thursday	Bedwetting (24 hrs: 9 AM-9 AM)	Overcrowding (24 hrs: 9 AM-9 AM)
Friday	Water deprivation (24 hrs: 9 AM-9 AM)	Food deprivation (24 hrs: 9 AM-9 AM)
Saturday	Cage tilting (24 hrs: 9 AM-9 AM)	Soiled cage (24 hrs: 9 AM-9 AM)
Sunday	Food deprivation (24 hrs: 9 AM-9 AM)	Overcrowding (24 hrs: 9 AM-9 AM)

SPT

The sucrose consumption test was conducted every week to assess anhedonia induced by the UCMS protocol, which is one of the core symptoms of major depression in humans [17]. Two bottles of 1% sucrose solution were placed in each cage for the first 24-hour period, and then one of the bottles containing 1% sucrose solution was replaced with tap water for the second 24-hour period. After the adaptation period, mice were deprived of water and food for 14 hours. The test was performed at 9:00 a.m. with the mice being placed individually in the cages and permitted free access to two bottles, one containing 100 ml of 1% sucrose solution and the other containing 100 ml of tap water. After three hours, the consumed volumes of sucrose solution and tap water were recorded. The reduced sucrose preference, which is used as an index of anhedonia, was calculated according to the following formula: 

$\frac{\text{sucrose consumption}}{\text{water consumption} + \text{sucrose consumption}} \times 100\%$

After four weeks of stress induction, a sucrose consumption test was carried out once a week during the treatment period (30 minutes after drug administration). Any change in sucrose consumption will be compared with the stressed control group.

OFT

The spontaneous exploratory behavior was measured in the OFT as described previously with minor modifications [18]. Decreased locomotor activity has been used as an index of low emotionality in mice. The open field was made of plywood (50 X 50 cm), in which the floor was divided into 25 (5 cm x 5 cm) identical sectors by white stripes. The field was further divided into central and peripheral sectors, where the central sector contained the nine central squares (3 cm x 3 cm), and the peripheral sector was the remaining squares. At the end of the sixth week, mice were placed individually in the center of the open field to explore freely for a five-minute session to measure locomotor activity. Horizontal locomotion (number of line crossings), rearing frequencies (the number of times an animal stood on its hind legs), and time spent in the center within the five-minute period were measured to evaluate the locomotor activity.

FST

The FST is based on the principle of behavioral despair [18]. When the mouse is placed in a container filled with water, it will first make efforts to escape, but eventually will exhibit immobility that may be considered to reflect a measure of behavioral despair.

The animals were transported in their home cages to the room at least 30 minutes prior to behavior testing in order to get them acclimatized to the testing environment. There was one session of six minutes long for each mouse, divided into pretest (the first two minutes) and test (the last four minutes). The animals were placed individually in a transparent Plexiglas cylinder (height: 30 cm, diameter: 20 cm) filled with tap water at 25°C, and the water depth was adjusted, usually up to 15 cm from the bottom. The mouse was placed in the water for six minutes, and the whole session was video recorded, and the duration of time spent immobile was measured. Immobility was defined as the absence of movement of the forelimb and hindlimb except that necessary to maintain the head above the water. The FST was performed on the sixth week, after 14 days of drug administration. 

TST

To assess the depression-like behavior, the TST test was performed as originally described by Steru et al. with minor modifications [20]. Accordingly, at the end of the sixth week, the animals were suspended 50 cm above the floor by means of an adhesive tape placed approximately 1 cm above the tip of the tail. The time during which mice remained immobile was quantified in seconds during a period of 6 minutes.

Statistical analysis

Data are presented as mean ± SD for each group (n = 6 animals). Group differences in behavioral outcomes (SPT, OFT, FST, and TST) were analyzed using one-way analysis of variance (ANOVA). Assumptions of normality and homogeneity of variance were verified before applying ANOVA. When the ANOVA indicated a significant overall effect, pairwise comparisons between treatment groups and the stressed control were performed using Tukey's Honestly Significant Difference (HSD) post-hoc test to adjust for multiple comparisons. A p-value < 0.05 was considered statistically significant. All statistical analyses were conducted using IBM SPSS Statistics software, version 21 (IBM Corp., Armonk, NY, USA).

## Results

**Figure 1:**
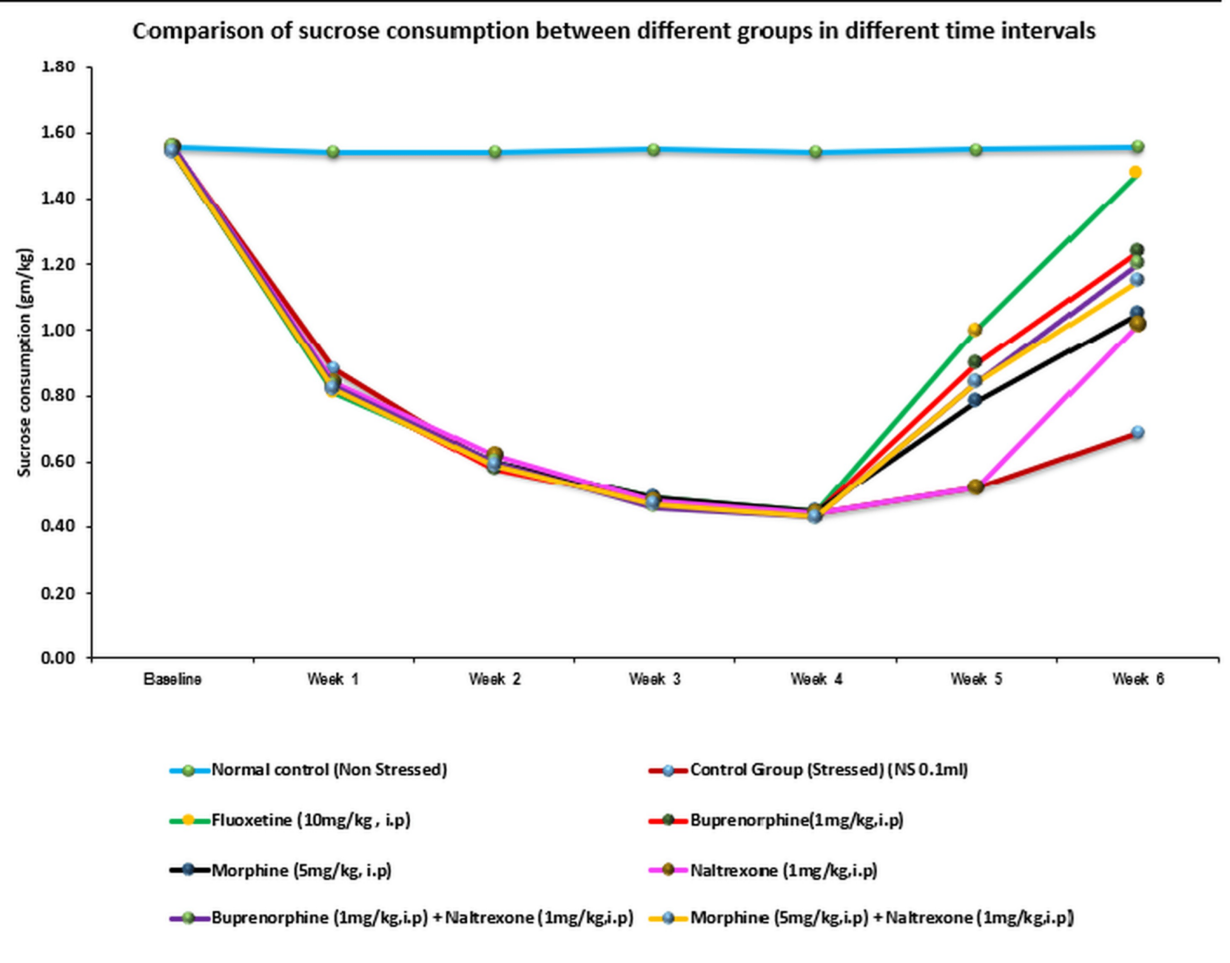
Sucrose consumption among different groups at different time intervals

In the present study, the baseline represents sucrose consumption before starting UCMS, while the first four weeks denote sucrose consumption during the induction of UCMS. The maximum reduction in sucrose intake was observed at the end of the four-week UCMS period, and the subsequent two weeks (weeks 5 and 6) reflect sucrose consumption during the treatment phase (Figure 1). The mean sucrose intake during the one-hour test is presented for weekly testing sessions in comparison to the control saline-treated group at the same time point. We have described the results only for the fifth and sixth weeks, as the drug treatment was initiated after the conclusion of the fourth week.

SPT 

The UCMS model caused a gradual decrease in the consumption of 1% sucrose solution in the stressed control group as compared to the normal control group (not exposed to the UCMS regime), with the maximum and significant decrease seen in the fourth week. The test applied was a one-way ANOVA followed by the Tukey HSD post-hoc test. The result of the one-way ANOVA showed that at Week 5, F(7,40) = 169.50, p < 0.0001, and at Week 6, F(7,40) = 145.77, p < 0.0001, meaning a highly significant difference exists between the groups.

In between-group comparisons with post-hoc tests, fluoxetine (10 mg/kg) showed a significant increase in sucrose consumption in both the fifth (p<0.0001) and sixth weeks (p<0.0001), and was statistically close to normal controls. Similarly, buprenorphine (1 mg/kg, IP) showed a significant increase in sucrose consumption in both weeks compared to stressed control animals (p < 0.0001). A similar significant effect was observed with the combination of buprenorphine and naltrexone, although the effect was less compared to buprenorphine alone. Morphine, naltrexone, and morphine+naltrexone showed no significant difference in sucrose consumption in both weeks in comparison to the stressed control group (Table 3).

**Table 3:** Effect of drug treatments on sucrose consumption (gm/kg) at week 5 and week 6 (Mean ± SD, n=6). P-value using one-way ANOVA followed by Tukey Honestly Significant Difference (HSD) post-hoc test. NS: normal saline

Group	Week 5 (Mean ± SD)	Week 5 vs. Normal control	Week 5 vs. Stressed control	Week 6 (Mean ± SD)	Week 6 vs. Normal control (p)	Week 6 vs. Stressed control(p)
Normal control (NS)	86.45 ± 3.27	–	–	86.38 ± 4.31	–	–
Stressed control (NS)	30.70 ± 4.90	<0.0001	–	31.46 ± 2.87	<0.0001	–
Fluoxetine	73.21 ± 5.56	0.0885	<0.0001	74.35 ± 3.52	0.2201	<0.0001
Buprenorphine	62.12 ± 4.11	0.00008	<0.0001	64.13 ± 4.37	0.0009	<0.0001
Morphine	34.83 ± 3.25	<0.0001	0.9822	35.19 ± 2.47	<0.0001	0.9933
Naltrexone	37.51 ± 3.34	<0.0001	0.688	39.06 ± 2.84	<0.0001	0.774
Morphine + Naltrexone	35.07 ± 3.77	<0.0001	0.913	36.96 ± 5.29	<0.0001	0.881
Buprenorphine + Naltrexone	51.01 ± 1.48	<0.0001	<0.0001	53.78 ± 6.10	<0.0001	<0.0001

OFT

UCMS exposure produced a highly significant reduction in locomotor activity, as reflected by horizontal locomotion (Table 4), rearing frequency (Table 5), and time spent in the center (Table 6), compared with the normal control group (p < 0.001). Chronic treatment with buprenorphine (1 mg/kg, i.p.) significantly increased all three locomotor measures compared with stressed controls at both week 5 and week 6 (p < 0.001), with a slightly greater effect at week 6. The buprenorphine + naltrexone combination also improved activity (p < 0.05), although the effect was less pronounced than buprenorphine alone. Fluoxetine (10 mg/kg, i.p.) produced robust improvements across all parameters (p < 0.001), compared to the stressed control group, approaching normal levels; by week 6, the difference vs. normal control was not significant. In contrast, morphine (5 mg/kg) caused a significant increase in only horizontal locomotion activity in week 5 (p=0.027) and in week 6 (p=0.005), while naltrexone and morphine + naltrexone did not significantly alter locomotor activity. 

**Table 4:** Effect of drug treatment on horizontal locomotion i.e. frequency of line crossing in five minutes (Mean ± SD) P-value using ANOVA followed by Tukey’s Honestly Significant Difference (HSD) post-hoc test

Groups	Week 5 (Mean ± SD)	Week 5 vs. Normal control	Week 5 vs. Stressed control	Week 6 (Mean ± SD)	Week 6 vs. Normal control	Week 6 vs. Stressed control
Normal control	76.5 ± 6.57	–	–	78.33 ± 9.09	–	–
Stressed control	50.33 ± 3.88	<0.001	–	52.83 ± 3.54	0.001	–
Fluoxetine	64.17 ± 6.43	0.005	<0.001	71.5 ± 6.72	0.138	0.003
Buprenorphine	62 ± 2.28	<0.001	0.003	68.33 ± 3.14	0.076	<0.001
Morphine	57.17 ± 2.32	<0.001	0.027	61.33 ± 2.66	0.008	0.005
Naltrexone	53.33 ± 3.56	<0.001	0.276	56.17 ± 3.66	0.007	0.029
Buprenorphine + Naltrexone	60.5 ± 2.66	<0.001	0.023	65.17 ± 2.32	0.028	<0.001
Morphine + Naltrexone	54.5 ± 2.74	<0.001	0.754	60.5 ± 3.62	0.003	0.095

**Table 5:** Effect of drug treatment on rearing frequency in five minutes (Mean ± SD) P-value using ANOVA followed by Tukey’s Honestly Significant Difference (HSD) post-hoc test

Groups	Week 5 (Mean ± SD)	Week 5 vs. Normal control	Week 5 vs. Stressed control	Week 6 (Mean ± SD)	Week 6 vs. Normal control	Week 6 vs. Stressed control
Normal Control	16.83 ±1.83	_	_	18.33±2.73	_	_
Stressed control	4±1.1	<0.001	_	6.00 ±1.26	<0.001	_
Fluoxetine	13.83±1.83	0.0452	<0.001	15.33±1.21	0.1375	<0.001
Buprenorphine	10.83 ±1.17	<0.001	<0.001	12.33 ±1.75	<0.001	<0.001
Morphine	4.17±0.75	<0.001	0.9990	6.17 ±1.17	<0.001	0.9990
Naltrexone	4.83±2.14	<0.001	0.8121	6.33±1.51	<0.001	0.9960
Morphine + Naltrexone	4.33±0.52	<0.001	0.9970	6.33 ±1.03	<0.001	0.9960
Buprenorphine + Naltrexone	7.83±1.17	<0.001	0.0018	9.33±1.03	<0.001	0.0023

**Table 6:** Effect of drug treatment on time spend in center(s) in five minutes (Mean ± SD) P-value using ANOVA followed by Tukey’s Honestly Significant Difference (HSD) post-hoc test.

Groups	Week 5 (Mean ± SD)	Week 5 vs. Normal control	Week 5 vs. Stressed control	Week 6 (Mean ± SD)	Week 6 vs. Normal control	Week 6 vs. Stressed control
Normal control	43.83 ±3.43	_	_	46.5±3.94	_	_
Stressed control	29±3.22	<0.001	_	30.5 ±2.43	<0.001	_
Fluoxetine	40.83±5.31	0.640	<0.001	43.5±1.87	0.012	<0.001
Buprenorphine	37.83 ±2.79	<0.001	<0.001	40.5±2.74	<0.001	<0.001
Morphine	30.67±2.80	<0.001	0.941	32.5±1.76	<0.001	0.632
Naltrexone	31.83±3.71	<0.001	0.340	34.33±1.75	<0.001	0.016
Morphine + Naltrexone	30.5±2.35	<0.001	0.984	31±2.61	<0.001	0.999
Buprenorphine + Naltrexone	34.83±1.6	<0.001	0.006	37.5±1.87	<0.001	<0.001

Overall, morphine, naltrexone, and the combination of morphine with naltrexone showed an insignificant increase in locomotor activity in both weeks as compared to the stressed group.

FST and TST

One-way ANOVA showed a highly significant treatment effect (P < 0.0001), indicating differences in the immobility times across groups. This was followed by Tukey’s HSD post hoc test. By the end of the sixth week, the stressed control group's immobility time had significantly increased (p<0.001) compared to the normal controls, confirming the depression model. Fluoxetine and buprenorphine (with or without naltrexone) did not differ from normal control, and they significantly decreased immobility time (P<0.001) when compared to stressed control, indicating strong antidepressant-like effects. Morphine, naltrexone, and morphine + naltrexone, on the other hand, also reduced immobility in comparison to stressed controls (P < 0.001), but their immobility times were still significantly higher than normal control, indicating a partial improvement (P < 0.021, P < 0.001, and P < 0.026 for morphine, naltrexone, and morphine + naltrexone, respectively) (Table 7). 

**Table 7:** Effect of drug treatment on immobility time (seconds) in Forced Swim Test (FST) in unpredictable chronic stressed mice (UCMS) P-value showing one-way ANOVA followed by Tukey's Honestly Significant Difference (HSD) post hoc test; Overall ANOVA: F(7,40) = 48.63, P < 0.0001 sec: seconds; UCMS: unpredictable chronic mild stress

Groups	Immobility time (sec) Mean ± SD	Vs. Normal control	Vs. Stressed control
Normal control	75.67 ± 3.93	_	_
Stressed control	120.5± 4.81		_
Fluoxetine	75.17± 3.76	0.800	<0.001
Buprenorphine	80.17± 5.38	1.000	<0.001
Morphine	89.83 ± 4.54	0.021	<0.001
Naltrexone	96.83± 8.45	<0.001	<0.001
Buprenorphine + Naltrexone	82.5 ± 5.65	0.943	<0.001
Morphine + Naltrexone	90± 6.07	0.026	<0.001

 In the TST model, the stressed control group exhibited significantly greater immobility than the normal control group and other treatment groups (F(7,40) = 47.71, p < 0.001). Fluoxetine and buprenorphine (with or without naltrexone) significantly reduced immobility, reaching values close to normal (no significant difference from normal control). Even with naltrexone and morphine alone, immobility was noticeably higher than in the normal control group, suggesting a less potent antidepressant-like effect (Table 8).

**Table 8:** Effect of drug treatment on Immobility time (seconds) in Tail Suspension Test (TST) in unpredictable chronic stressed mice (UCMS) P-value using one-way ANOVA followed by Tukey's Honestly Significant Difference (HSD) post hoc test; One-way ANOVA: F(7,40) = 47.71, p < 0.001 sec: seconds; UCMS: unpredictable chronic mild stress

Groups	Immobility time (sec) Mean ± SD	Vs. Normal control	Vs. Stressed control
Normal control	72.83 ±3.81	_	_
Stressed control	106.83 ± 7.83	<0.001	_
Fluoxetine	75.17 ± 6.31	0.996	<0.001
Buprenorphine	78.33 ± 3.01	0.812	<0.001
Morphine	86.17 ± 5.34	0.004	<0.001
Naltrexone	93.5 ± 3.45	<0.001	0.004
Buprenorphine + Naltrexone	80.5 ± 3.78	0.372	<0.001
Morphine + Naltrexone	88.33 ± 4.32	0.001	<0.001

## Discussion

This study provides compelling evidence that buprenorphine, alone and in combination with naltrexone, exerts robust antidepressant-like effects in the UCMS model. The findings extend previous reports of opioid modulation in depression and highlight KOR antagonism as a promising mechanism for antidepressant drug development.

Buprenorphine, a partial MOR agonist and KOR antagonist with additional activity at NOP receptors, significantly reversed UCMS-induced behavioral deficits across multiple domains, including sucrose preference, locomotor activity, and behavioral despair. Fluoxetine, the reference standard, produced comparable effects, supporting the validity of the model. In our study, the sucrose consumption was more with buprenorphine alone than with the buprenorphine-naltrexone combination. Although it was expected to be the other way round because of more enhanced kappa antagonism with the addition of naltrexone to buprenorphine. This implies that even though buprenorphine could be producing its effect through its actions at KORs, it does not rule out a possible role for other receptors like the delta opioid receptors (DOR) and the nociceptin receptor (NOP), since buprenorphine and its active metabolites bind to multiple opioid receptors [21]. Buprenorphine action as a low-efficacy partial agonist at NOP receptors, as well as norbuprenorphine’s potent agonist activity at DOR, could also contribute to its antidepressant effects [21]. In contrast, morphine showed limited efficacy in sucrose preference and locomotor assays but modestly reduced immobility in the TST, consistent with evidence that MOR agonism can transiently improve mood [22, 23]. Naltrexone alone was largely ineffective but, in combination with morphine, produced modest behavioral effects. These observations underscore the complex interplay of opioid receptor systems in regulating mood and stress responses.

The locomotor findings further support a role for KOR antagonism in restoring exploratory behavior suppressed by UCMS. Previous rodent studies have assessed the influence of morphine on locomotor effects and have demonstrated contrasting results depending on dose and time of administration, with both stimulant and depressive effects being reported [24, 25]. KOR activation exerts bidirectional influences on rearing and exploratory activity, with lower doses increasing this activity and higher doses decreasing it [26]. Buprenorphine’s consistent restoration of locomotor measures, therefore, likely reflects its KOR antagonist profile.

The results from the FST and TST strongly reinforce the antidepressant-like actions of buprenorphine. Reduced immobility was most marked with buprenorphine and fluoxetine, while the buprenorphine-naltrexone combination also produced significant benefit, albeit to a lesser degree. These findings align with prior reports that μ-agonists such as morphine and agmatine reduce immobility in despair paradigms, whereas KOR activation produces depressive-like behaviors [5, 27, 28]. The growing body of preclinical evidence consistently demonstrates that KOR antagonists reduce immobility, reverse anhedonia, and protect against stress-induced behavioral deficits [29, 30].

Taken together, these findings highlight buprenorphine as a potent modulator of depressive-like behaviors, with efficacy comparable to fluoxetine across multiple validated endpoints. While KOR antagonism appears to be the dominant mechanism, the engagement of additional opioid receptor systems may account for the breadth and robustness of buprenorphine’s effects. The addition of naltrexone may confer a safety advantage by reducing MOR-mediated abuse potential while retaining efficacy, supporting its translational potential as a safer opioid-based antidepressant strategy.

Limitations

This study has some important limitations. Only single doses of each compound were evaluated, precluding dose-response analysis. Neurochemical assays and histopathological assessments were not performed, limiting insight into receptor-level contributions and preventing conclusions regarding long-term safety. Behavioral tests, though widely validated, cannot fully recapitulate the multidimensional nature of human depression. Only male mice were used in our study, although the inclusion of female mice as well would have been better to understand sex-specific differences in depression and ensure the development of more effective, gender-specific treatments. Also, blinding was not done to eliminate potential observer bias. Finally, while the sample size was appropriate for behavioral pharmacology, the findings require replication in larger cohorts and across sexes to strengthen translational relevance.

## Conclusions

Buprenorphine demonstrated robust antidepressant-like efficacy in the UCMS model, with consistent benefits across sucrose preference, locomotor activity, and despair-based assays. Its effects are likely mediated primarily through KOR antagonism, though contributions from MOR, DOR, and NOP-receptor systems cannot be excluded. Co-administration with naltrexone preserved efficacy while potentially reducing abuse liability, supporting the translational promise of this strategy.

These findings reinforce KOR antagonism as a mechanistically novel and clinically relevant target for depression. Further research should delineate receptor-specific contributions, explore dose-response relationships, and assess neurochemical correlates to establish mechanistic validity. Clinical studies are warranted to evaluate the therapeutic potential and safety of buprenorphine-based regimens in treatment-resistant depression.
